# Authors, Ghosts, Damned Lies, and Statisticians

**DOI:** 10.1371/journal.pmed.0040034

**Published:** 2007-01-16

**Authors:** Elizabeth Wager

Since the earliest peer-reviewed publications of the late 17th century, conventions about the authorship of scientific papers—which were generally anonymous and attributed to the sponsor (in those days, usually the church or the king)—have evolved considerably [1]. Readers now want to know not only who paid for the research but also who did the work. Transparency (i.e., full disclosure) is now considered a moral responsibility, and many medical journals have introduced mechanisms for increasing transparency [2]. The International Committee of Medical Journal Editors (ICMJE) has also issued guidance on who qualifies for authorship [3], and their criteria have been updated and augmented several times in response to several authorship scandals [4]. Yet problems with authorship persist.

## A New Study on Ghost Authors

In a new study published in *PLoS Medicine*, Peter Gøtzsche and colleagues compared research protocols with study publications to examine the prevalence and nature of ghost authorship in 44 industry-initiated randomized trials [5]. They defined ghost authorship as occurring when anybody who wrote the protocol, did the statistical analysis, or wrote the manuscript was not listed as an author. Using this criterion of ghost authorship (which is based, loosely, on the ICMJE definition), they showed that 75% of the publications had ghost authors and, in all cases, the ghosts were statisticians.

This study is unique in using information from research protocols (which were provided by a Danish research ethics committee) to look for evidence of ghost authorship. Previous studies have either contacted named authors [6] or relied solely on disclosures in publications [7]. All methods have serious limitations. Named authors may be unwilling to admit that deserving colleagues have been omitted from the author list, and may also be reluctant to disclose the contributions of ghosts in the acknowledgments section. Protocols, written at the start of a study, might not record significant but unanticipated contributions or changes in personnel.

Readers want to know who paid for the research and who did the work.

Another unusual feature of Gøtzsche et al.'s study is the focus on statisticians. Most previous investigations and commentaries have focused on the involvement of medical writers. Gøtzsche et al. examined whether the person who wrote the protocol was included as an author on the subsequent publication. However, only five of the 44 protocols had named authors (in all cases, employees of the sponsoring company), and none of them were listed as authors or mentioned in the acknowledgments in the publication.

Professional medical writers are often involved in writing protocols and in preparing manuscripts for publication, but it has proved difficult to determine the frequency of such involvement [7,8]. Writers preparing protocols often work within the clinical or regulatory affairs departments of the sponsor, while those developing publications are more often freelance or employed by medical communications agencies. In other words, the individuals who work on protocols are usually not the same as those who prepare publications, so Gøtzsche and colleagues' study probably underestimated the number of writers who worked on the publications.

## Are Medical Writers and Statisticians Truly Authors?

The question of whether writers merit authorship if they are involved only at the publication stage of a study has not been resolved. Guidelines from the European Medical Writers Association state that such writers usually do not qualify for authorship although their role should be acknowledged [9]. The ICMJE criteria state that all authors should have made a substantial contribution not only to developing the manuscript but also to other aspects such as collecting, analysing, or interpreting the data [3]. It could be argued that the act of drafting a manuscript always involves an element of interpretation, yet many writers feel they do not fulfil the overarching principle that authors should be able to take public responsibility for the study.

However, the role of statisticians is slightly clearer. The ICMJE criteria state that involvement in data analysis (or study design) and contribution to the manuscript is a qualification for authorship. Gøtzsche and colleagues highlight what they call the “widespread practice of not including statisticians as authors for reports of randomised trials” and note that this “deprives readers of a key insight into the role of the company.” They urge editors to change from the traditional system of simply listing authors' names to listing individuals' specific contributions to research projects. Such a “contributorship” system was proposed almost ten years ago [10] and has been endorsed by the ICMJE [3], yet it has not been widely adopted outside the largest general medical journals.

Listing contributions has many advantages. It makes it easier for editors to detect ghost authors (if key roles such as data analysis or drafting a manuscript are missing from the list) and guest authors (who are listed despite having made little or no contribution to the study or publication).

## Implications of the Study

One clear implication of Gøtzsche et al.'s study is that the ICMJE authorship criteria are widely ignored. This may not be surprising in light of an earlier study that showed that 62% of a sample of 66 British academics disagreed with at least one aspect of the ICMJE criteria [11]. However, Gøtzsche and colleagues' study is the first to show how often statisticians are omitted from authorship lists. This omission might suggest either that the ICMJE criteria should be revised to reflect current thinking or that it should be more strongly enforced. But journal editors are often not well placed to detect authorship abuse (especially missing authors) and have a poor track record in terms of educating contributors about authorship criteria. A more pragmatic approach would be to adopt the contributorship system and let researchers and readers make up their own minds about who deserves to be listed.

In choosing the title for this article, I thought I'd adapt the aphorism that there are three types of lie: lies, damned lies, and statistics. But, writing a piece about authorship, I thought I should check to see who coined this phrase. Ironically, the authorship of this memorable quote is uncertain (although it has been attributed to Benjamin Disraeli and was used by Mark Twain) [12]. Whoever the author was, perhaps we should now admit that there are four types of lie: lies, damned lies, statistics, and the authorship lists of scientific papers, and that statisticians may be able to help prevent both the third and fourth types.

## Abbreviations

ICMJE: International Committee of Medical Journal Editors
